# Studies on Oligomer Metal Complexes Derived from Bisamic Acid of Pyromellitic Dianhydride and 4-Bromoaniline

**DOI:** 10.1155/2014/516274

**Published:** 2014-10-29

**Authors:** Yogesh S. Patel

**Affiliations:** Chemistry Department, Government Science College, Gandhinagar, Gujarat 382015, India

## Abstract

Novel oligomer metal complexes (**2a–f**) of the ligand 2,5-bis((4-bromophenyl)carbamoyl) terephthalic acid **(1)** were prepared using transition metal salts and characterized by various spectroscopic techniques. The geometry of oligomer metal complexes was carried out by electronic spectral analysis and magnetic measurement studies. Polymeric properties have also been carried out. Ligand was synthesized using pyromellitic dianhydride and 4-bromoaniline. It was duly characterized. All novel synthesized compounds **1** and **2a–f** were evaluated for their antibacterial and antifungal activity. The results showed significantly higher antibacterial and antifungal activity of oligomer metal complexes compared to the ligand.

## 1. Introduction

**Scheme 1:**
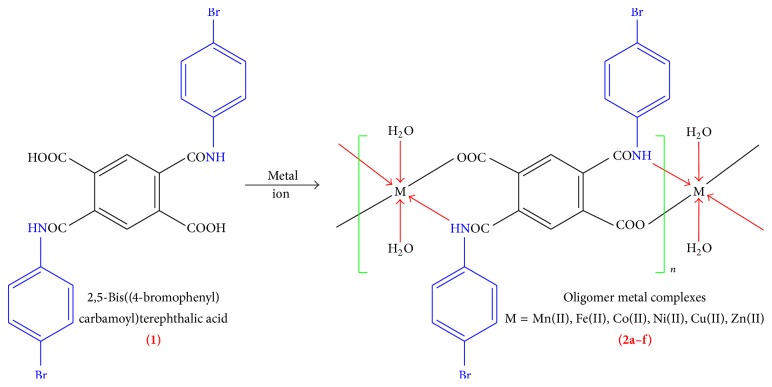
Synthetic route for the oligomer metal complexes.

The design and construction of polymeric metal complexes have received great attention [1–6]. The structures of metal complexes are depending upon the structure of the organic ligands, the coordinative geometry of metal ions, metal-ligand ratio, and other factors [7, 8]. Among various organic ligands, multicarboxylate ligands are often used to synthesize polymer metal complexes; for example, 1,2,4,5-benzene-tetra carboxylate, 3,3′,4,4′-biphenyl tetra carboxylic acid [9, 10], 1,1′-biphenyl-2,3′,3,4′-tetra carboxylic acid [11], and methylene diisophthalic acid [12] have been extensively used for the synthesis of various polymer metal complexes. On the other hand, the use of auxiliary N-containing ligands is also an effective method for the framework formation of polymer metal complexes owing to the fact that they can satisfy and even mediate the coordination needs of the metal center and consequently generate more meaningful architectures [13, 14]. The oligomer metal complex based on bisamic acid of pyromellitic dianhydride has not attracted any attention. Hence, initial work in this direction has been reported by us [15–18]. This prompted us to extend our work by using other auxiliary ligand such as 2,5-bis((4-bromophenyl) carbamoyl) terephthalic acid. With the aim of investigating the influence of ligand containing carboxylic and amide group (–O and –N containing ligand) on the frameworks of metal complexes, we have carried out the study for the reaction of metal (II) salts with novel bisamic acid.  Scheme 1 summarizes our synthetic approach to the synthesis of oligomer metal complexes using various metal (II) acetates, for example, Mn(II), Fe(II), Co(II), Ni(II), Cu(II), and Zn(II) metal ions. Ligand when incorporated with transition metal ions would produce a broad spectrum of antimicrobial property. The details of these procedures and the results obtained are discussed below.

## 2. Experimental

### 2.1. Materials and Measurements

All common reagents and solvents were used of analytical grade and were used without further purification. Alumina supported precoated silica gel 60 F254 thin layer chromatography (TLC) plates were purchased from the E. Merck (India) Limited, Mumbai, and were used to check purity of compounds and to study the progress of the reaction whereby TLC plates were illuminated under ultraviolet light (254 nm), evaluated in I_2_ vapors, and visualized by spraying with Dragendorff's reagent. Infrared spectra (FT-IR) were obtained from KBr pellets in the range of 4000–400 cm^−1^ with a Perkin Elmer spectrum GX spectrophotometer (FT-IR) instrument. ^1^H NMR and ^13^C NMR spectra were acquired at 400 MHz on a Bruker NMR spectrometer using DMSO-*d*_6_ (residual peak at *δ* ~2.5 or ~39.5 ppm, 300°K) as a solvent as well as TMS an internal reference standard. Microanalytical (C, N, H) data was obtained by using a Perkin Elmer 2400 CHN elemental analyzer. The solid diffuse electronic spectra were recorded on a Beckman DK-2A spectrophotometer with a solid reflectance attachment. MgO was employed as a reference. Magnetic moments [19] were determined by the Gouy method with mercury tetrathiocyanatocobaltate (II), [HgCo(NCS)_4_] as calibrant (*Xg* = 1644 × 10^−6^ cgs units at 20°C), by Citizen Balance (at room temperature). Molar susceptibilities were corrected using Pascal's constant [20]. The thermogravimetric studies were carried out with a model Perkin Elmer thermogravimetric analyzer at a heating rate of 10°C min^−1^ in the temperature range 50–700°C. The metal content of the oligomer metal complexes was carried by decomposing a weighed amount of each oligomer metal complexes with HClO_4_, H_2_SO_4_, and HNO_3_ (1 : 1.5 : 2.5) mixture followed by standard EDTA titration method [21]. Number average molecular weight ($\overset{-}{\text{Mn}}$) of oligomer metal complexes was determined by nonaqueous conductometric titration. It was carried out in pyridine solution against standard sodium methoxide in pyridine solution as titrant. The number average molecular weight of each sample was calculated according to method reported in the literature [22]. The melting point was checked by the standard open capillary method. In order to facilitate the correct structural assessment, that is, the coordination site, we have tried a lot to generate a crystal for single crystal X-ray analysis but we did not succeed. Hasanzadeh et al. [23] have reported this type of acid amide metal complex. So from the obtained data and reference article, interpretations become straightforward.

### 2.2. Synthesis of Ligand 2,5-Bis((4-bromophenyl) carbamoyl) Terephthalic Acid (1)

Adding a solution dropwise of 4-bromoaniline (24.40 g, 0.2 mol) to a stirred solution of pyromellitic dianhydride (21.813 g, 0.1 mol) and keeping the temperature of the medium close to 40–50°C for an hour (Scheme 1), thus obtained ensuing solution was poured into ice water in which the reaction product precipitated. The final white precipitates were filtered, washed, and purified by column chromatography. Physicochemical parameters and FT-IR spectral data were mentioned in Tables 1 and 2, respectively: ^1^H NMR (DMSO-*d*_6_, *δ* ppm): 10.87 (s, 2H,–COOH), 9.09 (s, 2H, –NH–), 8.48 (s, 2H, Ar–H), 7.66 (d, 4H, Ar–H), and 7.49 (d, 4H, Ar–H); ^13^C NMR (DMSO-*d*_6_, *δ* ppm): 121.3, 122.7, 126.8, 130.9, 134.3, 135.2, 136.8, 167.9, and 171.2.

### 2.3. Synthesis of Oligomer Metal Complexes (2a–f)

All oligomer metal complexes were synthesized by using equimolar amount of ligand and various metal (II) salts. To a warm clear solution of ligand (5.621 g, 0.01 mol) in 20 mL of dimethylsulphoxide add 0.1 M sodium hydroxide solution and pH about 7-8 was maintained. A pasty mass was observed. It was diluted with water to make a solution clear. To the above solution, metal (II) acetate solution (0.01 mol) was added with constant stirring and the pH of the reaction mixture was adjusted to 6-7 for** 2a**,** 2b**,** 2c**,** 2d**, and** 2f** and 4-5 for** 2e**. Thus oligomer metal complexes were separated out in the form of a suspension. It was digested on a water bath for 1 h, filtered, washed, and dried in air at room temperature. These oligomer metal complexes designated as** 2a–f** are insoluble in common organic solvents like methanol, ethanol, chloroform, acetone, and benzene.

### 2.4. Biological Activity

#### 2.4.1. Antibacterial Activity (*In Vitro*)

Compounds (**1** and** 2a–f**) were screened for* in vitro* antibacterial activity against Gram-positive bacterial strains (*Bacillus subtilis* (BS) and* Staphylococcus aureus* (SA)) and Gram-negative bacterial strains (*Salmonella typhimurium* (ST) and* Escherichia coli* (EC)) utilizing the agar diffusion assay [24, 25]. The wells were dug in the media with the help of a sterile metallic borer. Recommended concentration (100 *μ*L) of the test sample (1 mg/mL in DMSO) was introduced in the respective wells. Other wells supplemented with DMSO and reference antibacterial drug, ciprofloxacin, were served as negative and positive controls, respectively. The plates were incubated immediately at 37°C for 24 hours. Activity was determined by measuring the diameter of zones showing complete inhibition (mm). Growth inhibition was compared with the standard drug. In order to clarify any participating role of DMSO in the biological screening, separate studies were carried out with the solutions alone of DMSO and they showed no activity against any bacterial strains.

#### 2.4.2. Antifungal Activity (*In Vitro*)

Compounds (**1** and** 2a–f**) were also examined for antifungal activity against different fungal strains, that is,* Penicillium expansum* (PE),* Botryodiplodia theobromae* (BT),* Nigrospora sp*. (NS), and* Trichothecium sp*. (TS). The antifungal drug, ketoconazole, was used as a positive control. Antifungal screening for compounds (**1** and** 2a–f**) and positive control was performed at a recommended concentration. The fungal strains were grown and maintained on potato dextrose agar plates. The cultures of the fungi were purified by single spore isolation technique. Each compound (**1** and** 2a–f**) in DMSO solution was prepared for testing against spore germination of each fungus. The fungal culture plates were inoculated and incubated at 25 ± 2°C for 48 h. The plates were then observed and the diameters of the zone of inhibition (in mm) were measured. The percentage inhibition for fungi was calculated after five days using the formula given below:

(1) $$\begin{array}{c} \text{Percentage}\;\text{of}\;\text{inhibition}=\frac{100\left(X-Y\right)}{X}, \end{array}$$

where *X* is area of colony in control plate and *Y* is area of colony in test plate.

## 3. Results and Discussion

### 3.1. Synthesis of Ligand 2,5-Bis((4-bromophenyl) carbamoyl) Terephthalic Acid (1)

To the best of our knowledge, ligand (**1**) has not been reported previously. The characterization of the reaction product provided the first unambiguous proof of the successful synthesis of 2,5-bis((4-bromophenyl)carbamoyl)terephthalic acid. The FT-IR spectrum of ligand showed the most relevant peaks of the aromatic ring and 1,2,4,5-tetra substituted benzene ring, other than typical absorptions arising from the band at 3555 cm^−1^ and 1712 cm^−1^ for carboxylic acid and 3237 cm^−1^ and 1688 cm^−1^ for O=C–NH group [26]. In the ^1^H NMR spectroscopy, the signals in the range of 8.48, 7.66, and 7.49 ppm were ascribed to the protons of the aromatic rings. The singlet at 10.87 ppm was ascribed to the protons of carboxylic –OH group and a singlet at 9.09 ppm was attributed to the –NH proton of amide group, which was further confirmed by ^13^C NMR value, that is, 167.9 and 171.2 attributed to carboxylic carbon and amide carbon, respectively. The expected structure was thus clearly verified by the spectroscopic analysis which indicated moreover the absence of any detectable impurity, particularly of the two reagents used to prepare ligand.

### 3.2. Synthesis of Oligomer Metal Complexes

#### 3.2.1. Physical Properties

Elemental analysis of all polymer metal complexes was in good agreement with proposed structures. All polymer metal complexes exhibited 1 : 1 metal to ligand stoichiometry. The structures of oligomer metal complexes were consistent with the FT-IR, electronic spectra, and TGA. The geometry of the central metal ion was confirmed by electronic spectra and magnetic susceptibility measurements. The degrees of polymerization (DP) for all oligomer metal complexes are in the range of 5 to 6 (Table 1). All the data provides good evidence that the chelates are polymeric in nature. The suggested structure of the polymer metal complexes is shown in Scheme 1.

#### 3.2.2. Infrared Spectra

**Figure 1:**
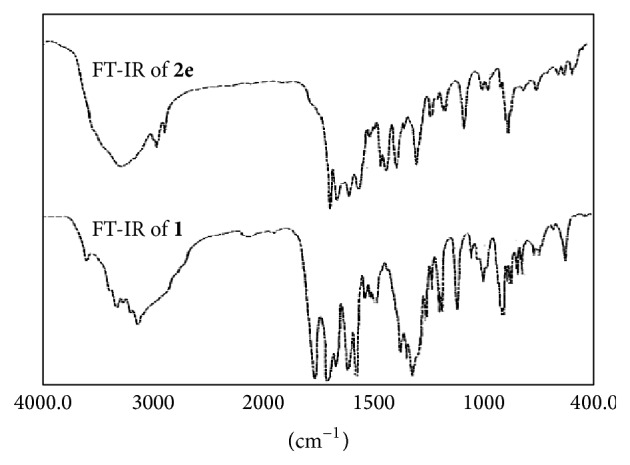
FT-IR of ligand and Cu-metal complex.

IR spectral bands of the ligand and its oligomer metal complexes suggest the formation of desired oligomer metal complexes and support their structure. Spectral features provide valuable information regarding the nature of functional group attached to the metal atom (Figure 1). In order to study the bonding mode of ligand to the oligomer metal complexes, the IR spectrum of free ligand was compared with the spectra of oligomer metal complexes (Table 2). Considerable differences to be expected were observed. The band at about 3555 cm^−1^ for carboxylic acid in ligand had virtually disappeared from the spectra of oligomer metal complexes. Oligomer metal complexes exhibit more broadened band in the region near 2980 cm^−1^ indicating the presence of coordinated water molecules [27]. The coordinated water in all the oligomer metal complexes presents different peaks at 980 cm^−1^ (rocking) and 770 cm^−1^ (wagging), whereas none of these vibrations appear in the spectra of uncoordinated ligands. A band at ~1640 cm^−1^ in free ligand is due to *ν*C–N vibration. The shifting of this group to lower frequency (~1610 cm^−1^) in the oligomer metal complexes when compared to free ligand suggested the coordination of metal ion through nitrogen atom of amide group [28]; it is expected that coordination of nitrogen to the metal atom would reduce the electron density in the amide link and thus lower the absorption [29]. A band at 1712 cm^−1^ is assigned to *ν*C=O stretching frequency in the spectrum of free ligand which is also shifted to lower frequency ranging from 1687 to 1694 cm^−1^ in all the oligomer metal complexes. This indicates the involvement of oxygen atom of hydroxyl group of –COOH group in bonding with metal ions [30]. New bands, which were not present in the spectrum of ligand, appeared in the spectra of oligomer metal complexes; for example, presence of sharp band in the region of 525–535 cm^−1^ can be assigned to *ν*M–N [30], which indicated the involvement of nitrogen in coordination. The medium intensity bands for *ν*M–O [31] have been observed at 625–635 cm^−1^ due to M–O coordination. The appearance of *ν*M–N and *ν*M–O vibrations supports the involvement of N and O atoms in complexation with metal ions under investigation. These overall data suggest that the amide-N and carboxylate-O groups are involved in coordination with the metal (II) ion in oligomer metal complexes. These features confirmed the proposed structure of oligomer metal complexes as shown in Scheme 1.

#### 3.2.3. Magnetic Moments and Electronic Spectral Data

The information regarding geometry of the oligomeric metal complexes were obtained from their electronic spectral data and magnetic moment values (Table 2). The diffuse electronic spectrum of the [Cu(L)(H_2_O)_2_]_*n*_ shows two broad bands around 15,949 cm^−1^ and 22,746 cm^−1^ due to the 2_T2g_ → 2_Eg_ transition while the second may be due to charge transfer, respectively. This suggests a distorted octahedral structure for the [Cu(L)(H_2_O)_2_]_*n*_ polymer which was further confirmed by its *μ*_eff_ value 1.99 B.M. The [Ni(L)(H_2_O)_2_]_*n*_ coordination polymer shows two absorption bands at 15,570 cm^−1^, 22,934 cm^−1^, and 9,851 cm^−1^ due to 3_A2g_ → 3_T1g_(F) and 3_A2g_ → 3_T1g_(P) and 3_A2g_ → 3_T2g_, respectively. The [Co(L)(H_2_O)_2_]_*n*_ polymer shows that two absorption bands, at 22,948 cm^−1^, 15,553 cm^−1^, and 9,845 cm^−1^ corresponding to 4_T1g_(F) →4_T1g_(P), 4_T1g_(F) →4_A2g_(F), and 4_T1g_(F) →4_T2g_(F) transitions, respectively, indicated an octahedral configuration for the [Ni(L)(H_2_O)_2_]_*n*_ and [Co(L)(H_2_O)_2_]_*n*_ polymers [32]. This configuration was further confirmed by its *μ*_eff_ values 2.87 B.M. and 4.25 B.M. The spectrum of [Fe(L)(H_2_O)_2_]_*n*_ shows bands at 36,062 cm^−1^ and 19,011 cm^−1^ assigned to the transitions 5_T2g_(F) →3_T1g_ and 5_T2g_(F) →3_Eg_ and its *μ*_eff_ 4.98 B.M. suggesting octahedral configuration. The spectrum of [Mn(L)(H_2_O)_2_]_*n*_ shows weak bands at 16,468, 17,769, and 23,140 cm^−1^ assigned to the transitions 6_A1g_ → 4_T1g_(4G), 6_A1g_ → 4_T2g_(4G), and 6_A1g_ → 4_A1g_, 4_Eg_, respectively, suggesting an octahedral structure for the [Mn(L)(H_2_O)_2_]_*n*_ polymer [33]. This configuration was further confirmed by its *μ*_eff_ value 5.54 B.M. As the spectrum of the [Zn(L)(H_2_O)_2_]_*n*_ polymer is not well interpreted, its *μ*_eff_ value shows that it is diamagnetic as expected. Magnetic moments *μ*_eff_ of all oligomer metal complexes revealed that all oligomers except Zn(II) metal ion polymer are paramagnetic while Zn(II) metal ion oligomer is diamagnetic.

#### 3.2.4. Thermal Analysis

**Figure 2:**
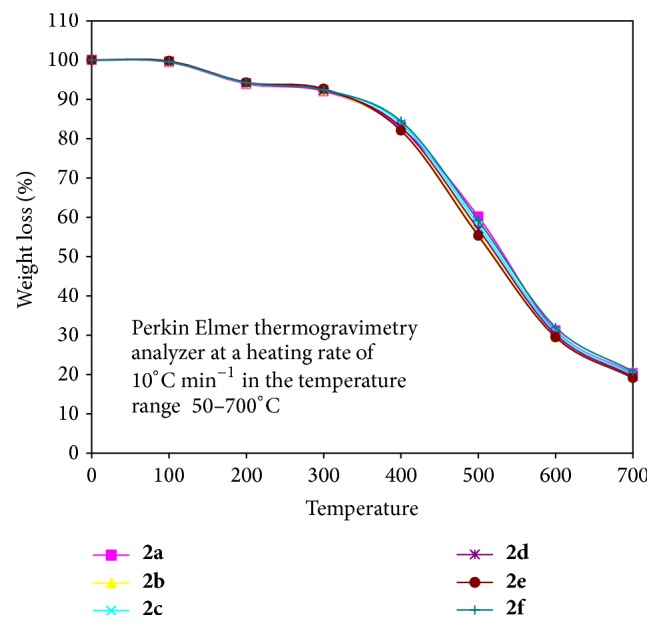
Thermogram of oligomer metal complexes.

The thermal behavior was investigated by Perkin Elmer TGA analyzer at a heating rate of 10°C min^−1^ in the temperature range 50–700°C under nitrogen which provides much information about the coordination compounds. In all the oligomer metal complexes, decomposition occurred in two steps (Figure 2). First step occurred between 100°C and 200°C which might be attributed to mass loss corresponding to water molecules. The value of weight loss during this step was consistent with theoretical value of two water molecules indicating that two water molecules were coordinated to the metal ion. Second step occurred between 200°C and 700°C which exhibits a mass loss corresponding to decomposition of ligand part in polymer. The weight loss of polymer metal complexes was noticeable between 300 and 600°C. The rate of degradation became maximum at a temperature between 400°C and 600°C. This may be due to acceleration by metal oxide which forms in situ. Each polymer loses about 80% of its weight when heated up to 700°C. On the basis of the relative decomposition (% weight loss) and the nature of thermogram, the oligomer metal complexes may be arranged in the order of their increasing stability as Cu < Fe < Ni < Co < Zn < Mn.

### 3.3. Biological Activity

#### 3.3.1. Antibacterial Activity

**Figure 3:**
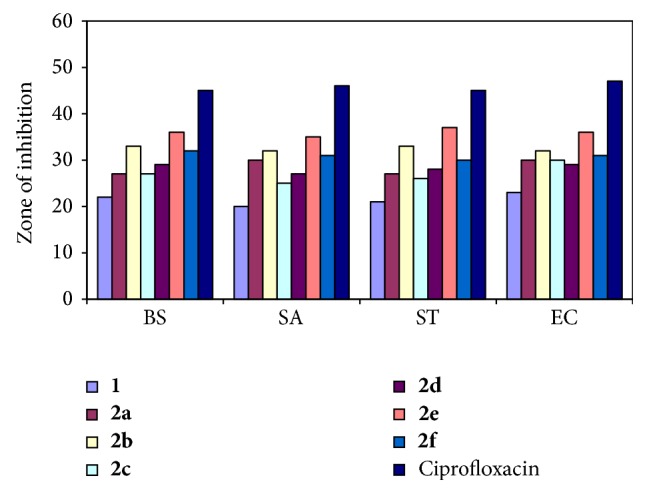
Antibacterial activity of ligand and its oligomer metal complexes.

Based on the data from the antibacterial studies against both Gram-positive and Gram-negative bacterial strains, the following observations can be made. All compounds (**1** and** 2a–f**) exhibited antibacterial activity against both Gram-positive and Gram-negative bacterial strains with zones of inhibition (ZOI) ranging from 20 mm to 37 mm (Figure 3).

Schiff base 2,5-bis((4-bromophenyl) carbamoyl)terephthalic acid was found less active than its metal complexes. Among the analogs** 2a–f**, compound** 2e** (ZOI_(BS)_ = 36 mm, ZOI_(SA)_ = 35 mm, ZOI_(ST)_ = 37 mm, and ZOI_(EC)_ = 36 mm) was identified as a potent antibacterial agent against all Gram-positive and Gram-negative bacterial strains. Compound** 2b** (ZOI_(BS)_ = 33 mm, ZOI_(SA)_ = 32 mm, ZOI_(ST)_ = 33 mm, and ZOI_(EC)_ = 32 mm) also had good antibacterial activity against bacterial strains. Compounds** 2a**,** 2c**,** 2d**, and** 2f** exhibited moderate antibacterial activity. Compounds** 1** and** 2a–f** exhibited less antibacterial activity as compared to standard antibiotic drug, ciprofloxacin (ZOI_(BS)_ = 45 mm, ZOI_(SA)_ = 46 mm, ZOI_(ST)_ = 45 mm, and ZOI_(EC)_ = 47 mm).

Comparative study of the growth inhibition zone values of Schiff base and its oligomer metal complexes indicated that the oligomer metal complexes exhibited higher antibacterial activity than free Schiff base (Figure 3). Such increased activity of the oligomer metal complexes can be explained on the basis of Overtone's concept and Tweedy's chelation theory [34]. According to Overtone's concept of cell permeability, the lipid membrane that surrounds the cell favors the passage of only lipid soluble materials due to which liposolubility is considered to be an important factor that controls the antimicrobial activity. Chelation reduces the polarity [35, 36] of the metal ion mainly because of the partial sharing of its positive charge with the donor groups and possibly the *π*-electron delocalization within the whole chelate ring system thus formed during coordination. This process of chelation thus increases the lipophilic nature of the central metal atom, which in turn favors its permeation through the lipoid layer of the membrane. This in turn is responsible for increasing the hydrophobic character and liposolubility of the molecule in crossing cell membrane of the microorganism and hence enhances the biological utilization ratio and activity of the testing drug/compound. The biological activity of compounds also depends on the nature of the ligand, concentration, lipophilicity, nature of metal ion, coordinating sites, and geometry of the complex.

#### 3.3.2. Antifungal Activity

**Figure 4:**
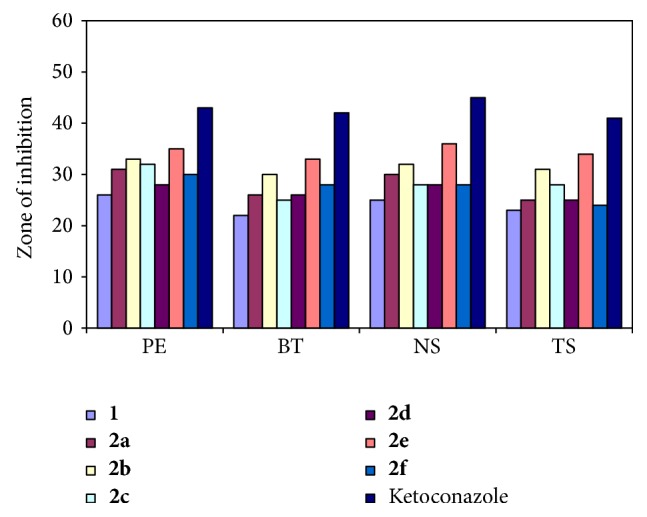
Antifungal activity of ligand and its oligomer metal complexes.

Based on the screening data from the antifungal studies, the following observations can be made. All compounds (**1** and** 2a–f**) exhibited antifungal activity against different fungal strains (Figure 4). Schiff base 2,5-bis((4-bromophenyl) carbamoyl)terephthalic acid was found less active than its oligomer metal complexes. Compound** 2e** (ZOI_(PE)_ = 35 mm, ZOI_(BT)_ = 33 mm, ZOI_(NS)_ = 36 mm, and ZOI_(TS)_ = 34 mm) was identified as more active against all fungal strains. Compound** 2b** (ZOI_(PE)_ = 33 mm, ZOI_(BT)_ = 30 mm, ZOI_(NS)_ = 32 mm, and ZOI_(TS)_ = 31 mm) also had good antifungal activity against fungal strains. Compounds** 2a**,** 2c**,** 2d**, and** 2f** exhibited moderate antifungal activity. Compounds** 1** and** 2a–f** exhibited less antifungal activity as compared to standard antibiotic drug, ketoconazole (ZOI_(PE)_ = 43 mm, ZOI_(BT)_ = 42 mm, ZOI_(NS)_ = 45 mm, and ZOI_(TS)_ = 41 mm).

## 4. Conclusions

Ligand and its oligomer metal complexes have been synthesized and were duly characterized by various spectroscopic techniques. The geometry of a central metal ion was confirmed by electronic spectra and magnetic susceptibility measurements. Antimicrobial activity of ligand and its oligomer metal complexes suggests that the complexes are more potent than the ligand.

## Figures and Tables

**Table 1:** Physicochemical parameters of the ligand and its oligomer metal complexes.

Empirical formula of compound		Empirical weight (gm)	Color	Yield %	M.P.^a^ (°C)	Elemental analysis calc. (found %)				*μ*_eff_ B.M.	${\bar{M}}_{n}$	DP
						C	H	N	M			
**1**	L C_22_H_14_Br_2_N_2_O_6_	562.16	Light yellow	65	160^b^	47.00 (46.89)	2.51 (2.46)	4.98 (4.92)	—	—	—	—

**2a**	[Mn–L–(H_2_O)_2_]_*n*_ C_22_H_16_Br_2_N_2_O_8_Mn	651.12	White	45	>250	40.58 (40.50)	2.48 (2.43)	4.30 (4.25)	8.44 (8.40)	5.54	3250	5

**2b**	[Fe–L–(H_2_O)_2_]_*n*_ C_22_H_16_Br_2_N_2_O_8_Fe	652.02	Light brown	58	>250	40.53 (40.45)	2.47 (2.42)	4.30 (4.23)	8.56 (8.50)	4.98	3901	6

**2c**	[Co–L–(H_2_O)_2_]_*n*_ C_22_H_16_Br_2_N_2_O_8_Co	655.11	Light pink	55	>250	40.33 (40.25)	2.46 (2.40)	4.28 (4.21)	9.00 (8.92)	4.25	3257	5

**2d**	[Ni–L–(H_2_O)_2_]_*n*_ C_22_H_16_Br_2_N_2_O_8_Ni	654.87	Light green	63	>250	40.35 (40.30)	2.46 (2.41)	4.28 (4.26)	8.96 (8.92)	2.87	3250	5

**2e**	[Cu–L–(H_2_O)_2_]_*n*_ C_22_H_16_Br_2_N_2_O_8_Cu	659.73	Green	70	>250	40.05 (40.00)	2.44 (2.38)	4.25 (4.21)	9.63 (9.58)	1.99	3941	6

**2f**	[Zn–L–(H_2_O)_2_]_*n*_ C_22_H_16_Br_2_N_2_O_8_Zn	661.56	White	45	>250	39.94 (39.88)	2.44 (2.39)	4.23 (4.17)	9.88 (9.82)	D	3312	5

^a^Melting points were checked by standard open capillary method and were found uncorrected; ^b^uncorrected.

*μ*_eff_  B.M.: magnetic moment, ${\bar{M}}_{n}$: number of average molecular weights, DP: degree of polymerization, and D: diamagnetic.

**Table 2:** FT-IR frequencies and electronic spectral data of the ligand and its oligomer metal complexes.

Compound		−COOH	−CONH	−OH	−C=O	−CONH	COO^−^	C–O	M–O	M–N	Electronic spectral data	
											cm^−1^	Transitions
**1**	L	3555	3237	—	1712	1688	1465	1047	—	—	—	—

**2a**	[Mn–L–(H_2_O)_2_]_*n*_	—	3212	2982	1692	1665	1453	1029	623	528	16,486, 17,769, 23,140	6_A1g_ → 4_T1g_(4G), 6_A1g_ → 4_T2g_(4G) 6_A1g_ → 4_A1g_, 4_Eg_

**2b**	[Fe–L–(H_2_O)_2_]_*n*_	—	3220	2979	1694	1673	1455	1026	628	531	19011 36062	5_T2g_(F) → 3_Eg_ 5_T2g_(F) → 3_T1g_

**2c**	[Co–L–(H_2_O)_2_]_*n*_	—	3211	2983	1690	1672	1447	1028	630	529	9,845 15,553 22,948	4_T1g_(F) → 4_T2g_(F) 4_T1g_(F) → 4_A2g_(F) 4_T1g_(F) → 4_T1g_(P)

**2d**	[Ni–L–(H_2_O)_2_]_*n*_	—	3214	2977	1695	1669	1449	1025	634	526	9,851 15,570 22,934	3_A2g_ → 3_T2g_ 3_A2g_ → 3_T1g_(F) 3_A2g_ → 3_T1g_(P)

**2e**	[Cu–L–(H_2_O)_2_]_*n*_	—	3225	2969	1689	1671	1450	1027	629	532	15,949 22,746	2_T2g_ → 2_Eg_ charge transfer

**2f**	[Zn–L–(H_2_O)_2_]_*n*_	—	3213	2973	1687	1668	1446	1026	632	527	—	—
